# Genetic Variability in the Italian Heavy Draught Horse from Pedigree Data and Genomic Information

**DOI:** 10.3390/ani10081310

**Published:** 2020-07-30

**Authors:** Enrico Mancin, Michela Ablondi, Roberto Mantovani, Giuseppe Pigozzi, Alberto Sabbioni, Cristina Sartori

**Affiliations:** 1 Department of Agronomy, Food, Natural resources, Animals and Environment—DAFNAE, University of Padova, Viale dell’Università 16, 35020 Legnaro (PD), Italy; enrico.mancin@phd.unipd.it (E.M.); cristina.sartori@unipd.it (C.S.); 2 Dipartimento di Scienze Medico-Veterinarie, University of Parma Via del Taglio 10, 43126 Parma, Italy; michela.ablondi@unipr.it (M.A.); alberto.sabbioni@unipr.it (A.S.); 3 Italian Heavy Draught Horse Breeders Association (ANACAITPR), 37068 Vigasio (VR), Italy; direzione@anacaitpr.it

**Keywords:** horse, genetic diversity, pedigree, genomic, SNP, inbreeding, ROH, effective population size, selection signatures, Italian Heavy Draught Horse

## Abstract

**Simple Summary:**

Genetic diversity has been investigated in Italian Heavy Draught horse (IHDH) using both a traditional and a genomic-based approach. The combined use of approaches has allowed depicting the complex history of the IHDH population, in which the progressive increase in inbreeding was counteracted by an increase in genetic variability when the population base was enlarged, also thanks to the contribution of French Breton stallions. A complex framework of the population structure was observed, with two subpopulations recognizable, which was likely to be due to breeding practices including the different use of French stallions to support the genetic variability in the breed. Some highly selected genomic regions were found and related to disease resistance, which was not specific for the two subpopulations. The population history and structure of IHDH has shown that despite the pretty small population size, genetic variability is still high in the breed, which does not yet require specific conservations programs.

**Abstract:**

This study aimed to investigate the genetic diversity in the Italian Heavy Horse Breed from pedigree and genomic data. Pedigree information for 64,917 individuals were used to assess inbreeding level, effective population size (Ne), and effective numbers of founders and ancestors (*f_a_/f_e_*). Genotypic information from SNP markers were available for 267 individuals of both sexes, and it allowed estimating genomic inbreeding in two methods (observed versus expected homozygosity and from ROH) to study the breed genomic structure and possible selection signatures. Pedigree and genomic inbreeding were greatly correlated (0.65 on average). The inbreeding trend increased over time, apart from periods in which the base population enlarged, when Ne increased also. Recent bottlenecks did not occur in the genome, as *f_a_/f_e_* have shown. The observed homozygosity results were on average lower than expected, which was probably due to the use of French Breton stallions to support the breed genetic variability. High homozygous regions suggested that inbreeding increased in different periods. Two subpopulations were distinguished, which was probably due to the different inclusion of French animals by breeders. Few selection signatures were found at the population level, with possible associations to disease resistance. The almost low inbreeding rate suggested that despite the small breed size, conservation actions are not yet required.

## 1. Introduction

The conservation and management of domestic animal genetic diversity has become a key issue in modern livestock breeding since the globalization of breeding programs [1]. Genetic diversity erosion, the increase in recessive allele frequency, and reduced performances in traits of breeding interest are the main consequences of mating among relatives [2]. The availability and completeness of population data are key aspects to assess genetic variability and develop strategic goals in biodiversity conservation [3]. In the last decades, traditional estimations based on pedigree data have been replaced or integrated with microsatellite data. Nowadays, the availability of high-density SNP chips has fostered the development of new tools to assess genetic diversity, offering a detailed picture of diversity across the genome [4]. The correlation between pedigree-based and molecular diversity depends on the completeness of pedigree information, as well as on the number and frequency of molecular markers. The number of markers is a key aspect, since too few are only able to reflect inbreeding at some (random) points along the genome. In contrast, pedigree-based diversity offers a global estimate [4]. Larger numbers of markers allow stronger correlations with pedigree inbreeding [5], despite the inability to obtain correlations equal to one since Mendelian sampling is ignored in pedigree-based inbreeding, and pedigree information are in some cases wrong or incomplete [4]. Therefore, the integration of pedigree-based and molecular information may provide a more meaningful overview on genetic diversity. [5].

A number of recent studies in livestock species have focused on the estimation of genetic diversity using both pedigree data and molecular information: in cattle [4,5], pigs [6], and horses. In horses, genetic diversity was estimated using pedigree data and microsatellites (e.g., in thoroughbred horses [7], Franches–Montagnes [8], and Belgian Draught horse [9]) and, more recently, using pedigree and high-density SNP information (Norik of Muran horses [10]).

The Italian Heavy Draught Horse (IHDH; Supplementary Figure S1) is a local Italian horse breed whose origin can be traced back to the formation of the Kingdom of Italy in 1861. The breed was established to support the development of a heavy horse for rapid draught purposes both in agriculture and field artillery [11]. The breed was settled by crossing Norfolk–Breton stallions (imported and used since 1911) from France (“Posthorse” or “Postier Breton”) with local heavy mares from the northeast of Italy. Since the institution of the studbook in 1927, a selection program was developed to obtain a homogeneous local population, even if French Bretons stallions were still introduced to keep genetic variability [11]. In the 1960s, the population size progressively decreased due to an increased mechanization in agriculture. Nonetheless, the breed survived thanks to the addition of the meat attitude (anacaitpr.it). Moreover, horses started to be selected also in Central and South Italy during the 1960s, also contributing to the official breeding nucleus since the end of the 1970s [11]. Breton’s stallions and mares were widely used in the 1980s and the early 1990s to increase the genetic variability toward meat production, but their use officially stopped in 2004. In those years, a complex situation occurred in IHDH breeding management: in North Italy Bretons stallions and mares were still used for breeding; in Central and South Italy, local mares were used only.

Nowadays, the meat attitude of the breed is still present, but the original heavy draught purpose has acquired increasing interest in recent years for agriculture, especially in organic farms, and for leisure activities such as team races [11,12]. The last official update (August 2019) of the DAD-IS database of the Food and Agriculture Organization-FAO (fao.org/dad-is/) reported a population size of 5137 individuals, including 353 stallions and 2962 mares in 792 studs. The population size has a slight decreasing trend, but the inbreeding rate is routinely monitored [11]. 

A selection program for meat and heavy draught attitudes based on linear type traits collected on young foals has been adopted in this breed [13]. The current IHDH individual is a bulky animal with an adult weighing 700–900 kg and an average wither height of 157 cm in males and 154 cm in females (anacaitpr.it; fao.org/dad-is/). The IHDH is nowadays spread across the whole country and reared both in stables (North Italy), and in feral or semi-feral conditions (Central and South Italy) [12].

Even though pedigree information has been recorded since the institution of the stud book (1927), an investigation of genetic diversity of the breed based on genealogical data has not been performed yet [11]. Except for individual inbreeding and average generation length [11], no additional information on genetic diversity parameters and on the effective population size were investigated before the present study. 

A pilot investigation on genetic variability based on molecular information was carried out using a panel of 23 microsatellite markers [14]. Here, a complex and fragmented structure of the population was observed, suggesting the occurrence of two subpopulations within IHDH breed and the importance of further analysis. Genotypic information on IHDH individuals was recently available, thanks to a national project for rural development in Italian horse breeds. These data may allow investigating both the population structure and the presence of genomic regions potentially under selection (also known as “selection signatures” [15,16]) in the breed. Recent developments in genomic methodologies have allowed further exploring the effects of a positive selection in the genome in terms of stretches of consecutive homozygous loci, which are also known as runs of homozygosity (ROH [17]).

Moving from these considerations, the present study aimed to perform an investigation of the genetic diversity of the IHDH breed by means of both pedigree and genotype data, including the investigation of a possible fragmentation of population structure looking at genotypic information and the search of selection signatures using ROH.

## 2. Materials and Methods

### 2.1. Data Structure

The study considered all studbook data available for the whole IHDH population updated at December 2019 and containing information since 1909 for an amount of 64,916 individuals after editing (Table 1). Editing included the exclusion of individuals without stud, birth date, or with an inconsistent birth date (original dataset included 66,122 individuals). An amount of 276 individuals were French Breton horses of both sexes, which were officially enrolled in the stud book of the breed. Animals of both sexes born in the last 10 years (since 2010) were chosen as the reference population (RefPop) for an amount of 14,016 individuals (not including French animals, since the latest French individual in pedigree was born in 2001).

An amount of genotypic information for 267 individuals of both sexes (Table 1) was collected in 2017–2019 thanks to a national project for rural development (PSRN Equinbio, D.M. 16/02/2018 n.5761) and made available by the National Breeders Association (ANACAITPR, anacaitpr.it). The animals selected for genotyping were all alive and widespread in the Italian territory, with both parents known and chosen as representative of the genetic variability of IHDH. An overview of all the data used in the study, both from pedigree and genotypes, is reported in Table 1.

### 2.2. Genotyping and Quality Control

Genotyping was performed using the commercial SNP panel “GGP Equine70k^®^” (Illumina, San Diego, CA, USA), including 65,157 markers spaced on average 40 kb. Genotype data were checked for the quality control (QC) measured using PLINK software, version 1.90 [18]. Specifically, SNP markers with a minor allele frequency (MAF) <0.01, loci with call rate ≤ 0.90, and extreme deviation from Hardy–Weinberg equilibrium (*p* < 10^−6^) were excluded in the further analyses. After quality control, an amount of 52,822 SNPs was retained. Then, the SNP markers were remapped from the previous reference genome EquCab2 using the most recent version of the Equine SNP map for 60 K SNPs, that is Eqcab3 [19], as described by Beeson et al. [20]. After the remapping, 50,919 SNPs were available. Finally, allosomes were not considered, due to the high differences between males and females allosomes, allowing retaining 50,720 SNPs for further analysis.

### 2.3. Pedigree Completeness and Diversity

The quality of pedigree data was evaluated in terms of pedigree completeness index (PC), which was calculated as the average of the percentages of known parents for each birth year in pedigree weighted for the number of newborns [21]. The pedigree completeness was manually calculated from pedigree data in a Microsoft Excel^TM^ spreadsheet. Moreover, the number of equivalent generations (EqGen) in the pedigree was calculated as the sum of the proportion of known ancestors over all the generations traced back, which was obtained by averaging the sum of all the (1/2)^k^ contributions of each known ancestor (with k = ancestor’s generation number, as 1 for parents 2 for grandparents, etc. [22]).

The average generation length (GL) was defined as the average age of parents at the birth of their offspring, which was computed by following the 4 gamete pathways: sire–son (GL_SS_), sire–daughter (GL_SD_), dam–son (GL_DS_), dam–daughter (GL_DD_) [23].

The genetic diversity of the IHDH population was investigated looking at the ancestors in the RefPop [24]. Ancestors were chosen on the basis of their marginal genetic contribution, which is the contribution not explained by other ancestors. The study also focused on the ancestors that are able to explain the 50% of the variability of the reference population (a50). Ancestors without known parents were considered as founders of the reference population.

The effective number of ancestors (*f_a_*) was considered as the equivalent number of ancestors, founders or not, explaining the whole genetic diversity of a population. The *f_a_* was defined considering the marginal genetic contribution q of a target ancestor *k*, as: $f_{a}=1/\sum_{k=1}^{f}q_{k}^{2}$ [24].

The effective number of founders (*f_e_*) was referred to as the number of equally contributing founders that would be expected to produce the same genetic diversity of the population. Considering the expected genetic contribution *p* of founder *j*, *f_e_* was: $f_{e}=1/\sum_{k=1}^{j}p_{j}^{2}$ [25].

The *f_a_*/*f_e_* ratio was computed to quantify the bottlenecks events that occurred over time in a population [26]. A ratio lower than 1 may be due to recent events of genetic drift such as bottlenecks [24].

### 2.4. Average Relatedness, Inbreeding, and Effective Population Size Using Pedigree Data

Pedigree analysis was also used to compute the average relatedness (AR) of individuals with the rest of the population, the individual inbreeding (F), and the effective population size (Ne) of IHDH.

The AR of each individual with the rest of the population was computed following Gutiérrez and Goyache [27].

The F was calculated for all individuals in the IHDH population using the traditional algorithm of Meuwissen and Luo (F_trad; [28]). The increase in inbreeding (ΔF) was calculated as regression over the equivalent generations traced back [29], with $\Delta F_{i}=1-\sqrt[{EqG_{i}-1}]{\left(1-F_{i}\right)}$, where F_i_ is the value of traditional inbreeding (F_trad) for individual i, and EqGi is the number of individual equivalent generations traced back. Another approach for computing F was also tried to recover the incomplete pedigrees, as proposed by VanRaden [30]. This approach calculates an individual inbreeding (F_rec) using a recursive algorithm developed by Aguilar and Misztal [21], assigning to individuals with missing parents an inbreeding equal to the average value for newborns in the same birth year.

The effective population size of IHDH from pedigree data (Ne_ped_) was defined as the dimension of an idealized population rising from the variation in F between generations. The parameter was computed using the method of Gutiérrez et al. [29], calculating Ne from the individual increase in inbreeding, as ${\mathrm{Ne}}_{\mathrm{ped}}=1/\left(2\bar{\Delta F}\right)$, with ΔF being the increase in inbreeding estimated from F_trad.

Parameters were computed on the Refpop and also by grouping the individuals by decade of birth (e.g., 1990s, 2000s, 2010s is equal to Refpop).

All parameters except pedigree completeness index and recursive inbreeding (F_rec) were computed using ENDOG software, ver. 4.8 [27]. Recursive inbreeding was obtained using INBUPGF90 [21].

### 2.5. Inbreeding and Effective Population Size Using Genomic Data

Genomic inbreeding was calculated in two methods: (1) in terms of observed versus expected number of homozygous genotypes, using Plink software [18], and (2) using the information from ROH. The first estimate was obtained using the specification—--het, which computes F coefficient (F_het) as the difference between the observed homozygotes count (H_obs_) and the expected homozygotes count (H_exp_).

The second method was based on runs of homozygosity (ROH) [31]. The ROH segments were identified using the DetectRUNS [32] package in R software [33] and defined as follows: at least 15 SNPs in a run; a minimum run length of 500 kb; a maximum distance of 1000 kb between successive SNPs in a window; a lower density limit of 1 SNP per 100 kb [34]; a maximum of 1 missing SNP and 1 heterozygous SNP in a run [35]. The ROH segments were divided into the following five classes of length: 0–1 Mb, 1–2 Mb, 2–4 Mb, 4–8 Mb, and l >8 Mb. Descriptive statistics were computed for each length class. 

The genomic inbreeding coefficient (F_roh) was calculated following the method described in [18]: $F\_\mathrm{roh}=\Sigma \frac{L_{\mathrm{ROH}}}{L_{\mathrm{AUTO}}}$, where L_ROH_ is the length of ROHs in each individual and *L_AUTO_* is the length of the autosomal genome covered by SNPs, which was equal to 2276 Gb. 

The effective population size of IHDH breed arising from genotypic information (Ne_gen_) was estimated using the SNeP v.1.1 program [36], which is able to estimate the trends of the historical effective population size trajectories from SNP data. The Ne_gen_ was estimated from the linkage disequilibrium [37]. The Sved and Feldman’s mutation rate modifier [38] was used to compute the recombination rate, as done in other studies in horses [39], and a correction for sample size was performed for unphased genotypes. The average genome-wide recombination rate was set to 1.24 cM/Mb [39]. A minimum and a maximum distance between SNP pairs were set as 0.5 Mb and 26 Mb, respectively, to evaluate recent N_e_ reduction (linkage disequilibrium-LD over greater recombinant distances) and past N_e_ reduction (shorter distances provide information on more distant times in the past). The thresholds depend on both the SNP panel and on the target species. The minimum distance was chosen as equal to 0.5 Mb to take out markers too closely located, and the maximum distance was set as the length of the shortest chromosome in the species. The effective population size was estimated for the current and for the last 18 generations considering the above-mentioned history of the breed.

### 2.6. Investigation of Population Structure Using Genomic Information

A previous study in IHDH using microsatellites data [14] showed a fragmented population structure. Therefore, we further investigated this aspect based on genomic information and using two different but complementary approaches. At first, the possible occurrence of subpopulation was explored by inferring the ancestry proportions of individuals based on K potential components. The sparse Non-negative Matrix Factorization algorithm sNMF [40] implemented in the R/Bioconductor package LEA, version 3.3.0 [41] was used. A number of K values from 1 to 10 were examined using default parameters, and for each value of K, 7 runs were computed, looking at literature and computational demands. Then, the best fitted run (Bayesian Information Criterion, or BIC [42]) was retained. The number of ancestral populations was determined by comparing the cross-entropy values for each K, and choosing the K that minimized the cross entropy [40]. Then, 25 runs were performed for the chosen value (K = 2), and the best fitted run was retained to assign individuals to each of the 2 subpopulations.

Then, the structure of these two subpopulations was investigated using the second approach. To visualize the population structure of IHDH horses based on the two sNMF subpopulations, we performed a high-resolution network analysis in the Netview package in R [43]. Genome-wide allele-sharing distances were calculated as one minus the average proportion of shared alleles, based on Identical by state (IBS) relationships in PLINK software, version 1.90 [18]. From the pair-wise distances between all individuals, a fully connected population network was created using an unsupervised network clustering method called Super Paramagnetic Clustering (spc) [44]. In this network, individuals are considered as nodes, and connections between a pair of individuals are considered as edges. The spc computes such a network using an algorithm requiring the specification of the maximum number of nearest neighbours (k-NN) that an individual can have. A k-NN = 10 was set as the default value, as suggested by previous applications [43,44,45].

Then, the 2 subpopulations found using sNMF were alternatively considered as a reference population to search for the ancestors explaining the variability of each subpopulation [27]. Then, the f_a_/f_e_ ratio and the number of ancestors describing the 50% of population variability (a50) were considered for further understanding the reason behind the presence of potential subpopulations. Finally, subpopulations were compared in terms of individual inbreeding level, considering the *F_roh* as an inbreeding coefficient.

### 2.7. Detection of Selection Signatures Using ROH

Signatures of selections were investigated by looking at the ROH shared among the majority of the animals. For this purpose, “DetectRUNS” [32] was used to detect ROH islands using a similar approach than in other studies on horses [46]. Putative ROH islands were firstly determined considering the overlapping homozygous regions within more than 60% of the IHDH genotyped individuals. The EqCab3 genomic coordinates of these regions were used to retrieve lists of annotations of the candidate genes. Information on the genes included in the ROH were obtained by enquiry through the Biomart web interface of the ENSEMBL data bank [47] and the UCSC genome browser platform [48]. To further investigate the function of the potential under selection regions, we decided to consider a more stringent threshold of selected individuals, as also elsewhere in horses [49]. Therefore, we compared the ROH shared in more than 70% of the animals with quantitative trait loci (QTLs) previously reported in the Horse Quantitative Trait Locus Database (Horse QTLdb) of the Animal Quantitative Trait Loci (QTL) Database (Animal QTLdb) [50]. Following the criteria reported above, putative ROH islands were searched for the 2 subpopulations found by the sNMF analysis, and compared with the ones that referred to the whole population.

## 3. Results

### 3.1. Pedigree Completeness and Diversity

Pedigree completeness and population genetic diversity arising from pedigree data are reported in Table 2. Pedigree completeness measured as PC index was 87.5%, increasing to 98.2% (data not shown) in the reference population defined as animals born from 2010. The pedigree completeness increased over time, but a quick reduction in completeness happened at the end of 1970s (average PC = 57.6% in years 1978–1981; Supplementary Figure S2), when the enlargement of the stud book with animals born out of the northeast of Italy occurred (see Introduction).

The number of individual equivalent generations (*EqGen*) traced back in pedigree was 3.25 ± 1.41 for the whole population, increasing to 4.49 ± 0.92 in the Refpop (data not shown). The number of EqGen progressively increased along the population history (Supplementary Figure S2), apart between the end of the 1960s and 1980s, consistently with the enlargement of the IHDH population.

An average generation length of 8.94 ± 4.02 was found in the IHDH breed, which was higher than 9 years in the dam pathways (GL_DS_ and GL_DD_).

The parameters from probability of gene origin (i.e., founders, ancestors, and related parameters) are also reported in Table 2. The ancestors/founders ratio was 0.93, reflecting the high completeness of the pedigree. The IHDH reference population genetic variability was explained by 1673 ancestors; nevertheless, 50% of the variability was explained by only the 1.1% of them (19 individuals). Among them, 15 horses were French Breton stallions, and the first 7 positions, explaining 30.8% of the genetic variability, were occupied only by French animals (data not shown). The present genetic diversity of the population was measured by the effective number of founders *f_e_*, that is 56, for a *f_a_/f_e_* ratio of 0.91, suggesting that important bottlenecks (expected with a *f_a_/f_e_* below 0.5 [51]) have not occurred. 

### 3.2. Average Relatedness, Inbreeding, and Effective Population Size Using Pedigree Data

**Figure 1:**
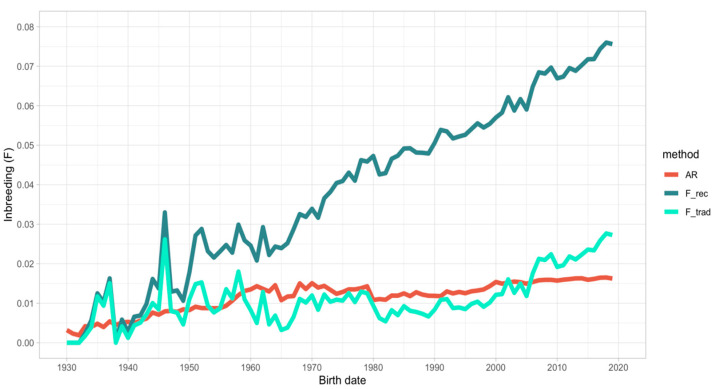
Trend of average relatedness (AR), traditional (F_trad), and recursive (F_rec) inbreeding in the last 90 years.

Average relatedness (AR), traditional (F_trad) and recursive (F_rec) inbreeding, individual increase in inbreeding (ΔF), and effective population size (Ne_ped_) from pedigree data computed for each decade of birth and for the whole IHDH population are reported in Table 3. The trends of AR, F_trad, and F_rec over 90 years of IHDH history (since 1930) are presented in Figure 1.

The average relatedness of IHDH individuals was 1.39% in the whole pedigree and 1.61% in the reference population (individuals born in the 2010s). Average relatedness increased over time in the IHDH population, apart from a couple of moments (in the middle of the 1960s and in the 1980s), roughly corresponding to moments of expansion of the breed.

Similarly, the population inbreeding, which was 1.39% for the whole pedigree and 2.28% for the reference population, measured following Mewissen and Luo [28] (F_trad), largely varied at the beginning of the IHDH history to move toward a slight increase over time (ΔF = 0.51% for the whole population). The only decrease was shown in the beginning of the 1960s and in 1980, consistently with the reduction of AR mentioned above. In addition, the individual increase in inbreeding (ΔF) was the lowest in that decade. The current population (Refpop) reported values of F_trad and ΔF of 2.28% and 0.67%, respectively. Accounting for the incomplete genealogies in pedigree using the recursive algorithm for inbreeding (F_rec; [21]) allowed obtaining a roughly constant increase in individual inbreeding over time, up to a value of 5.57% for the whole population and 7.08% for the Refpop. In addition, the rate of inbreeding variation was greater using this approach for computing inbreeding (data not shown), but looking at the last 40 years (since 1980), the F_trad and F_rec variation were roughly parallel (Figure 1).

The effective population size (Ne_ped_) computed from the individual increase in inbreeding [29] was 97.1 in the whole pedigree and 74.3 for the Refpop. A slight decrease of Ne_ped_ was observed over time, apart for the periods in which the base population of IHDH enlarged. Two noteworthy growths of Ne_ped_ happened in the 1960s and 1980s, consistently with the decrease of F_trad and ΔF.

### 3.3. Inbreeding and Effective Population Size Using Genomic Data

An average of 35,630 observed homozygous genotypes was observed in genotyped individuals, whereas an average of 35,707 homozygous genotypes was expected (Supplementary Table S1), which were used at an individual level to estimate the inbreeding level based on observed and expected homozygosity. A total of 72,231 ROHs were found among the 267 horses analyzed in this study, with an average of 270 ROHs per individual. The majority of ROHs were shorter than 2 Mb with 66.0% being shorter than 1 Mb and 23.0% being between 1 and 2 Mb length (Table 4). The proportion of ROHs longer than 8 Mb was equal to 1.0%, with an average length of 13.61 Mb. A total of 250 horses exhibit ROH in the longest ROH class.

**Figure 2:**
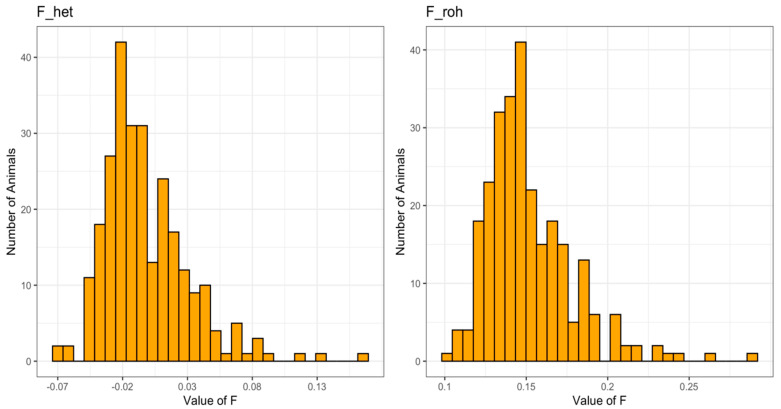
Distribution of genomic inbreeding coefficients based on the comparison of observed and expected homozygous genotypes (F_het) and on ROH (F_roh) in genotyped individuals.

The observed versus expected homozygous genotypes and the ROH allowed for all the genotyped individuals to estimate genomic inbreeding coefficients (F_het and F_roh, respectively). The F values calculated by the above-mentioned methods showed a similar trend distribution, but they differed for a constant value of 15.9 ± 0.1% (Figure 2). Only two individuals showed an excess of homozygosity with inbreeding levels (F_roh) above 25%.

**Figure 3:**
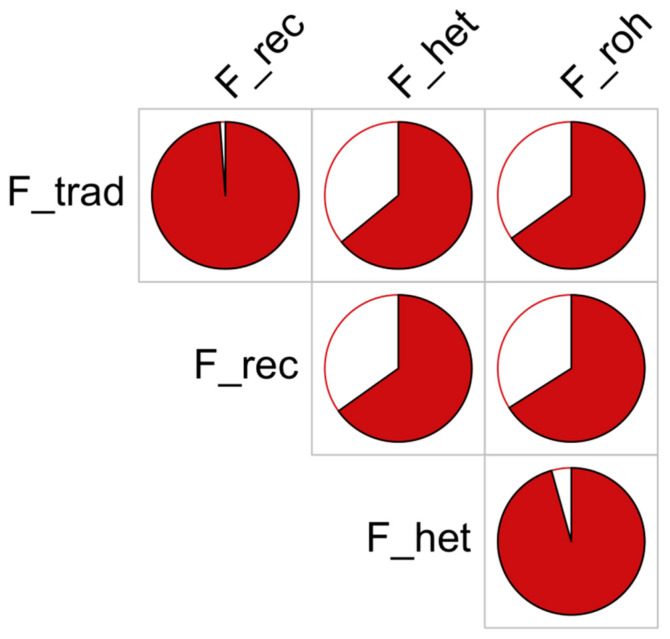
Comparison of inbreeding values obtained using different methods (definitions of methods in the text). The pie charts report the correlations (in red) between each pair of individual inbreeding values obtained with different methods.

The descriptive statistics of inbreeding based on the four methods used in this study can be found in Table 5. The average inbreeding varied considerably across the methods adopted in this study ranging from 15.36% for the F_roh to −0.51% for the F_het. Nevertheless, moderate to high correlations were found among the different applied methods, which are shown in Table 5 and Figure 3. The highest correlation was found between the two methods using pedigree data (98.8%; F_trad, F_rec) followed by the correlation between F_het and F_roh (96.0%). The lowest correlation was instead found between F_trad and F_het (64.1%). Overall, the pedigree and genomic inbreeding results greatly correlated (65% on average).

**Figure 4:**
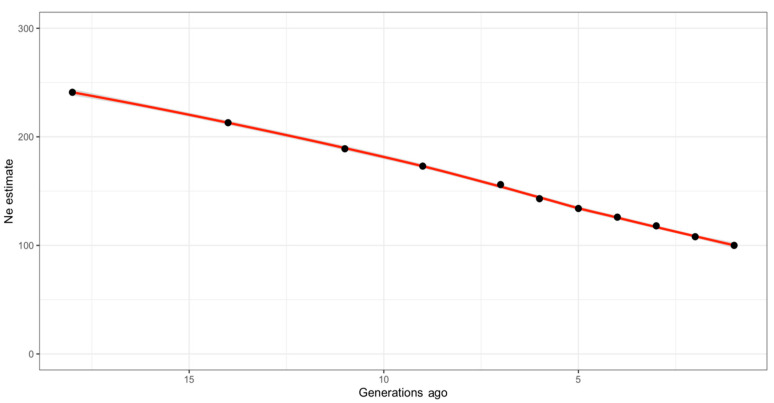
Estimation of genomic effective population size (Ne) traced back to 18 generations ago.

The effective population size of the IHDH horse breed declined over time, as shown in Figure 4 and also reported in Supplementary Table S2, including the measure of linkage disequilibrium at each generation. Effective population size was estimated to be approximately 100 horses one generation ago. The estimated Ne 18 generations ago was instead around 241 horses (Figure 4).

### 3.4. Investigation of Population Structure Using Genomic Information

**Figure 5:**
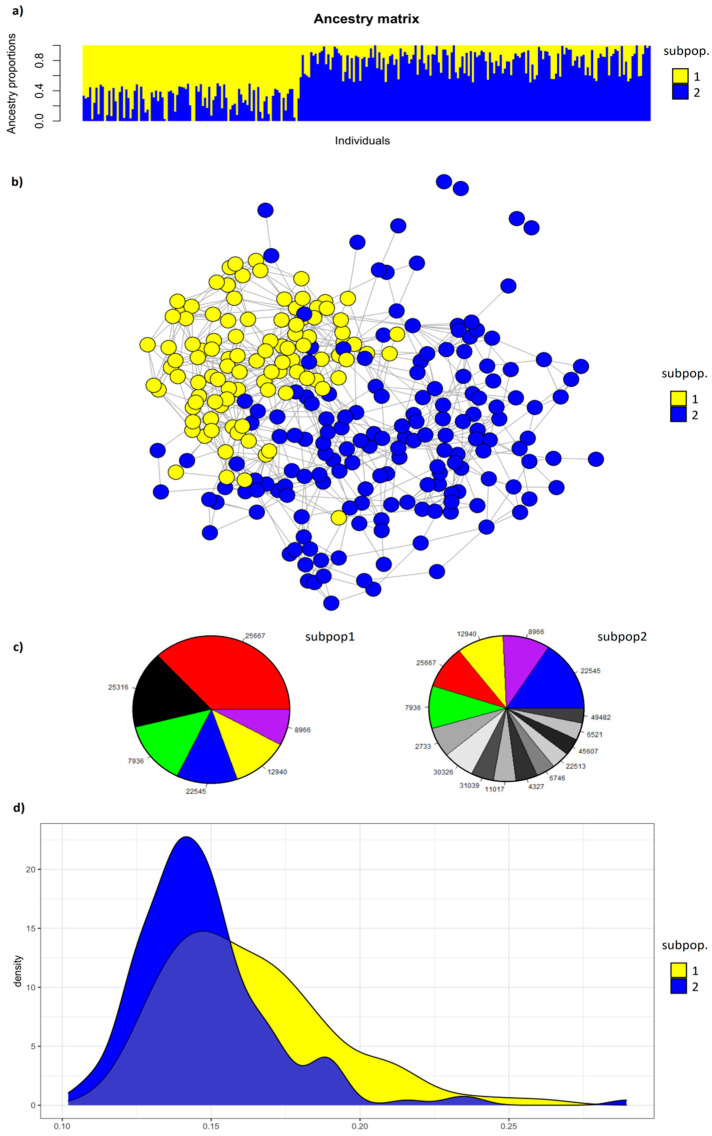
Population structure of Italian Heavy Draught Horse breed. From the top to the bottom: (**a**) clustering output for K = 2 (*subpop1* and *subpop2*) for genotyped individuals. Each individual is represented by one vertical line with the proportion of assignment to each cluster (ancestry proportion) on the y-axis; (**b**) population structure network for genotyped individuals, considering *subpop1* and *subpop2* resulted in (**a**). Individuals are nodes, and their relative genetic distances are represented by lines; (**c**) ancestors explaining 50% of the genetic diversity for *subpop1* and *subpop2*. The same ancestors in the two groups have the same color and label, and the other ancestors are in a gray–black scale; (**d**) plot of individual genomic inbreeding (F_roh) in *subpop1* and *subpop2.*

The values of BIC [42] for the best fitted runs from K = 1 to 10 (Supplementary Figure S3) have shown a better fitting for values of K = 1, but K = 2 showed just a slightly lower fitting. The value of K = 2 is be a putative number of different clusters in the whole population, and it was chosen to discuss the population structure of the breed. The ancestry proportion for each individual at values of K = 2 (Figure 5a) showed a similar pattern of the previous work on IHDH breed using microsatellites [14], suggesting a possible fragmentation of population structure with 2 subpopulations (*subpop1* and *subpop2*), respectively of 102 and 155 individuals.

A similar conclusion was found looking at the population structure network built based on genotyped individuals (Figure 5b). The individuals belonging to *subpop1* and *subpop2* segregated into these two subpopulations with few individuals only misplaced by the algorithm. Looking at the graph, the individuals of *subpop1* were more closely connected to each other than the individuals of *subpop2*. Moreover, four individuals, belonging to *subpop2*, were less related with the other ones, being not connected with any of the other individuals.

From a genealogical point of view, the genetic diversity of the two subpopulations was explained by 109 ancestors for *subpop1* and 195 for *subpop2*, including 26 (that is the 24%) and 48 (24% as well) French Breton animals, respectively. The effective numbers of founders (*f_e_*) were 18 and 42, creating *f_a_/f_e_* ratios of 0.89 and 0.93, respectively (data not shown). Furthermore, *subpop1* and *subpop2* had 6 and 15 ancestors, respectively, explaining 50% of the genetic diversity (a50; Figure 5c). Five of the 6 a50 for *subpop1* were also among the a50 of *subpop2*, but with a different rank. The a50 horse explaining most of the genetic diversity of *subpop1* (19.0%) (Supplementary Table S3) was a French Breton stallion born in 1996, which explained in *subpop2* 4.9% of the genetic diversity. The second position among the 50 of *subpop1* was covered by a local mare explaining 8.6% of the genetic diversity and not included among the ancestors of *subpop2*. The first 5 positions of a50 for *subpop2* were covered by the a50 of *subpop1* apart from the above-mentioned local mare. The remaining ancestors were individuals not included in a50 of *subpop1*, and in 3 cases also not included among all the ancestors of *subpop1* (data not shown). Five of the 6 a50 horses for *subpop1* are French Norfolk–Breton stallions, as well as 11 of the 15 a50 horses for *subpop2* (Supplementary Table S3). Only 41 among the 304 ancestors in total were shared among the two subpopulations (data not shown).

The distribution of inbreeding coefficient (Figure 5d), measured as *F_roh* on genotyped animals, partially overlapped for *subpop1* and *subpop2*, with higher values, on average, for *subpop1* compared to *subup2* (16.3 ± 2.8% for *subpop1* versus 14.7 ± 2.4% for *subpop2*; data not shown).

### 3.5. Detection of Selection Signatures Using ROH

A total of six ROH islands were shared in more than 60% of the horses and overlapped with 106 protein-coding genes. Two ROH islands were located on ECA3, one was located on ECA10, two were located on ECA11, and one was located on ECA15 (Supplementary Table S4). Among them, the two ROH islands located on ECA3 and the one located on ECA11 were shared in more than 70% of the horses, and these were considered for investigating gene functionality. Table 6 shows the genomic coordinates of the ROH islands and the annotated protein-coding genes. The longest ROH island was located on ECA3 and has a length equal to 815.4 kb, whereas the shortest was located on ECA11 (length = 98.3 kb). A total of 33 protein-coding genes were located within the three ROHs islands shared in more than 70% of the horses. The two ROHs islands on ECA3 located between position 35,477,778 and 36,946,465 overlapped with known QTLs for Insect bite hypersensitivity [52,53], white markings [54], and guttural pouch tympani disease [55]. The ROH island on ECA11 did not overlap with known QTLs. When considering the two subpopulations separately, a total of 23 ROH islands shared in more than 60% of the within-subpopulation individuals were found (Supplementary Table S5). A total of 12 ROH islands were in common among the two subpopulations. The remaining ones were unique for the subpopulation one except in one case. The genomic coordinates of the genomic elements located within the ROH islands found per subpopulation are presented in the Supplementary Table S5.

## 4. Discussion

The present study intended to use both pedigree and molecular information to depict the IHDH genetic diversity as clearly as possible. Since the population size of the IHDH breed has decreased consistently over the last few years, the evaluation and knowledge of the current genetic diversity is essential to eventually perform genetic conservation actions [2].

The pedigree data of the IHDH breed are likely to offer reliable information, since the pedigree completeness is high, especially in the last decade, as shown in the reference population. Likewise, the equivalent number of generations higher than 3 suggested a rather complete pedigree [22]. The definition of a decade (2010–2019) as a reference population is mainly because the breed is late maturing [11], and the average generation length is close to 9 years [11]. Long generation intervals were found also in other horse breeds, such as Bardigiano (8.47; [56]), Italian Haflinger (9.71; [57]), and Lusitano (10.52; [58]). The variation of pedigree completeness, inbreeding level, and effective population size over time reflected the IHDH population history. Lower inbreeding levels and thus an increased population size were found when the population basis of the breed has been enlarged. This happened in the 1960s, after the enlargement of the breeding area to the center and south of Italy, and in the 1980s, when the horses from the new breeding areas started to be used for selection purposes (see Introduction). In the 1980s, the selective bases of the population enlarged also due to the increased number of French Breton stallions and mares used for breeding. 

French animals were only 0.42% of the individuals in pedigree, but their contribution is massive. The analysis of ancestors and founders showed that 78.9% of the ancestors explaining the 50% of the population is represented by French horses. Nevertheless, the IHDH population did not experience recent events of bottlenecks, as suggested by the ratio among the effective number of ancestors and founders (*f_a_/f_e_*; [24] as important bottlenecks are expected with a *f_a_/f_e_* below 0.5 [51]). 

The massive introduction of new animals along IHDH history has led to a low and rather constant value of average relatedness over time. This later aspect will potentially allow a better control of the inbreeding in the long term [59]. Inbreeding has remained low over time, undergoing a reduction when the breed was enlarged (the new individuals had a pedigree-based inbreeding of zero (F_trad, [28]). A second pedigree-based inbreeding coefficient (F_rec) was considered able to recover incomplete pedigree information [21], but it has to be noticed that the latter methodology could overestimate the inbreeding level. This is because the founders’ animals up to 2004 (the year in which the introduction of French horses stopped) are not related. The F_rec was able to determine a pretty constant increment of inbreeding (ΔF) over time. However, this inbreeding rate is largely lower than the threshold of ΔF = 1% that FAO recommends for small populations [60]. In addition, the effective population size from pedigree data was definitely higher than the recommended threshold of 50 individuals per generation [60] along the whole history of IHDH.

Molecular information was able to complement the results found from pedigree data. The Ne based on linkage disequilibrium information from SNPs allowed tracing an historical trend of the effective population size, back to the beginning of the history of the breed [36]. A similar approach was used to detect the ancestral effective population size also in other horse breeds, such as in Finnish horse [58]. The current population size estimated with this approach is a bit higher than what was measured with pedigree information. As a matter of fact, a low level of homozygosity was found in IDHD genotyped animals, which was even lower than what was expected from SNP information. As a consequence, the inbreeding level computed as the difference between the observed and expected homozygous genotypes (F_het) was lower than zero in 168 of the 267 genotyped individuals. However, highly correlated estimates, roughly differing for a constant value, were obtained between F_het and F_roh. Many studies in various species, including horses [35,45], have demonstrated that ROH are a feasible source of information to describe genomic inbreeding [61]. Moreover, the identification of ROH segments can be useful to investigate complex population histories and structures [61], commonly assuming that long consecutive homozygous segments are the result of identical haplotypes from common ancestors [62]. Therefore, they are useful to estimate inbreeding coefficients for individuals with incomplete pedigree information. The high correlation between pedigree-based inbreeding and genomic inbreeding coefficients is the result of both the pedigree completeness of IHDH (reliable information on individual inbreeding needs high-quality pedigree data [62]), and of the density of SNP information [4]. Moreover, ROH segments of different lengths reflect inbreeding events that occurred in different time frames [61]. Most of the IHDH genotyped individuals show ROH in all the length classes considered, suggesting the occurring of inbreeding events both in the recent history of the breed (likely to be due after the 1980s) and in the past decades (1940s). 

The use of French Breton in the breed was reflected by the fragmented structure of the population at the genotype level, which is a trend previously found using microsatellites [14]. Genotype data showed that one subpopulation, here referred to as *subpop1*, showed highly “connected” individuals with a genomic inbreeding measured from ROH (F_roh) on average a bit higher than in individuals who belonged to the other subpopulation (called *subpop2*). A complex population structure with subpopulations identified using clustering methods based on genomic data was also found in Lipizzan horse [45] and in Noriker [63]. However, pedigree information showed a certain degree of overlap between the two subpopulations. Mostly, the same ancestors (all French Norfolk–Breton stallions) explained most of the genetic diversity of the subpopulation to which they refer but with different amounts of explained genetic variability. The extensive use of a single stallion contributing to 19% of the whole genetic diversity of *subpop1* and a wide contribution of a local mare not included among the ancestors of the other subpopulation, which highly contributed to determining the separation of the two subpopulations. Looking at the history of the breed, it is possible to note that many ancestors of *subpop2* came from the Regional Equestrian Breeding Centre of Ferrara that was used to introduce Breton stallions from France for breeding purposes until this practice was officially stopped for the whole IHDH breed. However, the two subpopulations have never been recognized or treated as different in IHDH breeding management, and also the selection pressures to which they were subdued were the same.

Runs of homozygosity have been widely used in animal genetics to detect both within-breed loss of genetic diversity and the potential signature of selections [4,64,65]. Overlapping homozygous regions, highly shared among individuals belonging to the same population, are thought to be potential signs of selection around a target locus. Several examples of ROH analyses in horses are available, and they address key aspects of the breed history and selection pressure [17,35,46,61,63,66]. In this study, we detected three ROH islands shared in over the 70% of the animals, highlighting potential signatures of selection in two regions on ECA3 and one region on ECA11. The ROH islands located on ECA3 overlapped with known QTLs for white markings which are indeed highly present in this breed (Supplementary Figure S1). These two ROH islands overlapped also with QTLs for two disease-related traits: the insect bite hypersensitivity and the guttural pouch tympani; therefore, we cannot rule out the hypothesis that those regions might be under selection due to their association with disease resistance. In addition, those two regions highly overlapped with the potential selection signatures found in two other draught horse breeds: the Noriker horse breed [46] and the Muran horse breed [67]. Therefore, we can suggest that some of the genes located in those two regions might have an important function for draught horse. The current breeding program in the IHDH is mainly designed for meat production, but we did not find any signs of potential selection related to this trait. The only molecular information linked to meat production was found in a previous study finding some associations between the morphological traits evaluated for meat and myostatin gene polymorphism [4]. Moreover, fewer ROH islands were detected in the IHDH breed if compared to European breeds mainly selected for sport disciplines [35]. A possible explanation might be related to the multiple aims currently present in the IHDH breeding program, including meat production and several types of leisure activities. The lack of significant differences from the ROH island analysis based on the two subpopulations suggested that the difference found from the population structure analyses is mainly due to ancestors’ effects rather than different breeding purposes [46,67]. A further use of high-density SNP panels (600 K), with a 10-fold number of SNPs, or a different marker panel that is more appropriate for this breed could maybe allow identifying additional ROH, if present. However, a high density would not be useful for better clarifying the occurrence of two subpopulations more clearly. Only the handling of the animals over time would eventually favor the definition of subgroups or not, depending on the breeding purpose. 

## 5. Conclusions

To conclude, the present study has allowed investigating the genetic diversity in Italian Heavy Horse Breed looking at both pedigree (the whole stud book data) and molecular information (SNP markers of 267 horses). Pedigree information were almost complete and allowed reliable estimations of inbreeding values, resulting in medium to high correlations with genomic inbreeding. The inbreeding trends increased over time, apart from the time points when the base population of the breed increased (between the 1950s and 1960s, and in the 1980s) and thus also the effective population size. The effective numbers of founders and ancestors showed that recent bottlenecks did not occur in the IHDH genome. Genomic information showed an observed homozygosity that was on average lower than the expected homozygosity, which was likely due to the use of French Breton stallions as breeding animals. High homozygous regions were found in the IHDH genome, suggesting that inbreeding increased in different moments along the breed history. The complex history of IHDH was reflected in a fragmented population structure: two subpopulations are suggested for genotyped individuals, which was probably due to the effect of the use of French Breton stallions. However, selection pressure did not differ in the two subpopulations, and few selection signatures were found at the population level, with a possible association to disease resistance and not with the two selection aims of the breed: meat and heavy draught. The complex history of IHDH breed, characterized by events of reduction and expansion of the breeding nucleus, including the introduction of foreign animals for breeding, has determined an almost low inbreeding level and rate, suggesting that despite the rather small size of the breed, conservation actions are not yet necessary. Notwithstanding, the individuation of two subpopulations could help for breeding decisions, with a practice of optimal contribution selection policies aimed at maximizing the genetic gain without a great increase of individual inbreeding.

## Figures and Tables

**Table 1:** Structure of pedigree and genotypic data used in the study.

	Pedigree Data	Genotypic Data
Individuals	64,916	267
Males	29,163	146
Females	35,753	121
First year	1909	2003
Last year	2019	2019
Native	64,640	267
Bretons	276	0
Reference population ^1^	14,016	-
- Males	6558	-
- Females	7458	-

^1^ Only for pedigree data.

**Table 2:** Pedigree completeness and population genetic diversity in Italian Heavy Draught Horse from pedigree data. Number of equivalent generations and generation lengths are shown as mean ± standard deviation. Generation intervals, ancestors, founders, and related statistics refer to the reference populations, as individuals born in the last 10 years.

Parameter	Values
Pedigree completeness (PC; %)	87.5%
Number of equivalent generations (*EqGen*)	3.25 ± 1.41
Generation length sire–sire (GL_SS_)	8.74 ± 3.79
Generation length sire–daughter (GL_SD_)	8.71 ± 3.77
Generation length dam–son (GL_DS_)	9.15 ± 4.21
Generation length dam–daughter (GL_DD_)	9.15 ± 4.24
Ancestors	1673
Founders	1795
ancestors explaining 50% of population (a50)	19
-Norfolk–Bretons animals	15
effective number of ancestors (*f_a_*)	51
effective number of founders (*f_e_*)	56
*f_a_/f_e_* ratio	0.91

**Table 3:** Average relatedness (AR), traditional (F_trad) and recursive (F_rec) inbreeding, individual increase in inbreeding (ΔF), and effective population size (Ne_ped_) from pedigree data for each decade of birth and for the total Italian Heavy Draught Breed population.

Decade	≤1930	1940	1950	1960	1970	1980	1990	2000	2010 ^1^	Total
Individuals	924	1034	1737	1437	2585	8700	16,512	17,971	14,016	64,916
AR (%)	0.37	0.69	1.00	1.32	1.37	1.19	1.29	1.54	1.61	1.39
F_trad (%)	0.40	0.77	1.21	0.75	1.14	0.75	0.96	1.61	2.28	1.39
F_rec (%)	0.47	1.19	2.47	2.65	4.08	4.73	5.36	6.27	7.08	5.57
ΔF (%)	0.55	0.75	0.69	0.39	0.63	0.31	0.39	0.57	0.67	0.51
Ne_ped_	90.4	66.6	72.2	128.5	79.3	159.7	129.1	88.2	74.3	97.1

^1^ This decade is also the reference population.

**Table 4:** Runs of homozygosity (ROH) of different length classes in individual genotypes.

Length Class (Mb)	N Individuals	N_ROH_^1^	% N_ROH_	S_ROH_^2^	L_ROH_^3^
0–1	267	47,597	0.66	178.27	0.68
1–2	267	16,686	0.23	62.49	1.33
2–4	267	4892	0.07	18.32	2.75
4–8	267	1995	0.03	7.47	5.50
>8	250	1061	0.01	4.24	13.61

^1^ N_ROH_: whole number of ROH; ^2^ S_ROH_: average ROH number; ^3^ L_ROH_: average length of ROH (Mb).

**Table 5:** Descriptive statistics of inbreeding values obtained using all the methods considered in the paper, from both pedigree (F_trad, F_rec) and genomic (F_het, F_roh) data.

Inbreeding ^1^	Mean	SD	Min	Max
F_trad (%)	1.66	2.01	0.00	10.91
F_rec (%)	6.49	1.96	4.41	15.57
F_het (%)	−0.51	3.32	−7.31	16.23
F_roh (%)	15.36	2.68	10.20	28.94

^1^ Definitions of the methods for inbreeding are in the text.

**Table 6:** ROH islands shared in over 70% of the Italian Heavy Draught horse (IHDH) horses with genomic coordinates and annotated genes located within each ROH island and related quantitative trait loci (QTLs) in footnotes.

ECA ^1^	Start (Bp)	End (Bp)	Length (Kb)	Annotated Genes
3 ^2^	35,477,778	36,008,377	530.6	*ZNF469, ZFPM1,ZC3H18, IL17C, CYBA, MVD, SNAI3, RNF166, CTU2, PIEZO1*
3 ^3^	36,131,080	36,946,465	815.4	*CBFA2T3, ACSF3, CDH15,SLC22A31, ANKRD11, SPG7, RPL13, CPNE7, DPEP1, CHMP1A, SPATA33, CDK10, SPATA2L, VPS9D1, ZNF276, FANCA, SPIRE2, TCF25*
11	24,275,465	24,373,721	98.3	*SP2, PNPO, CBX1, SNX11, SKAP1*

^1^ ECA: *Equus caballus* chromosome; ^2^ QTLs: Insect bite hypersensitivity; White markings; Guttural pouch tympany; ^3^ QTLs: White markings; Guttural pouch tympany.

## Supplementary Materials

The following are available online at https://www.mdpi.com/2076-2615/10/8/1310/s1, and included in the Supplementary Materials file: Supplementary Figure S1. Mares and foal of Italian Heavy Draught Horse; Supplementary Figure S2. Number of Equivalent Generations and pedigree completeness over years in Italian Heavy Draught Horse population; Supplementary Table S1. Descriptive statistics of homozygosity (observed: Ho_obs; expected: Ho_exp; total: Ho_tot) in 267 genotyped individuals of Italian Heavy Draught Horse based on the number of homozygous genotypes; Supplementary Figure S3. Values of BIC obtained by analyzing values of K from 1 to 10, corresponding to the same amount of clusters defining the proportion of ancestry in the 267 genotyped individuals; Supplementary Table S2. Estimation of genomic effective population size (Ne) traced back to 18 generations ago (Gen. ago). The linkage disequilibrium estimation, adjusted for sampling bias was also included, as well as the relative standard deviation; Supplementary Table S3. Ancestors explaining 50% of the genetic diversity in the two subpopulations (*subpop1* and *subpop2*) recognized in IHDH looking at 267 genotyped individuals; Supplementary Table S4. Genes coordinates, names, and types included in the ROH islands shared in over 60% of the IHDH horses with genomic information; Supplementary Table S5. Genes coordinates, names and types included in the ROH islands shared in over 60% of the two subpopulations of IHDH horses with genomic information recognized after clustering analysis
