# Supplementary material for: Genetic Variability in the Italian Heavy Draught Horse from Pedigree Data and Genomic Information
Source: Animals (Basel). 2020 Jul 30;10(8):1310. doi: 10.3390/ani10081310 (PMC7460293; doi:10.3390/ani10081310)
Supplement: Supplementary file 1 [file animals-10-01310-s001.pdf]

Supplementary material for manuscript:

Genetic variability in the Italian Heavy Draught Horse from pedigree data and genomic information.

Enrico Mancin†, Michela Ablondi†, Roberto Mantovani*, Giuseppe Pigozzi, Alberto Sabbioni and Cristina Sartori

** Correspondence: roberto.mantovani@unipd.it
† These two Authors equally contributed to the work

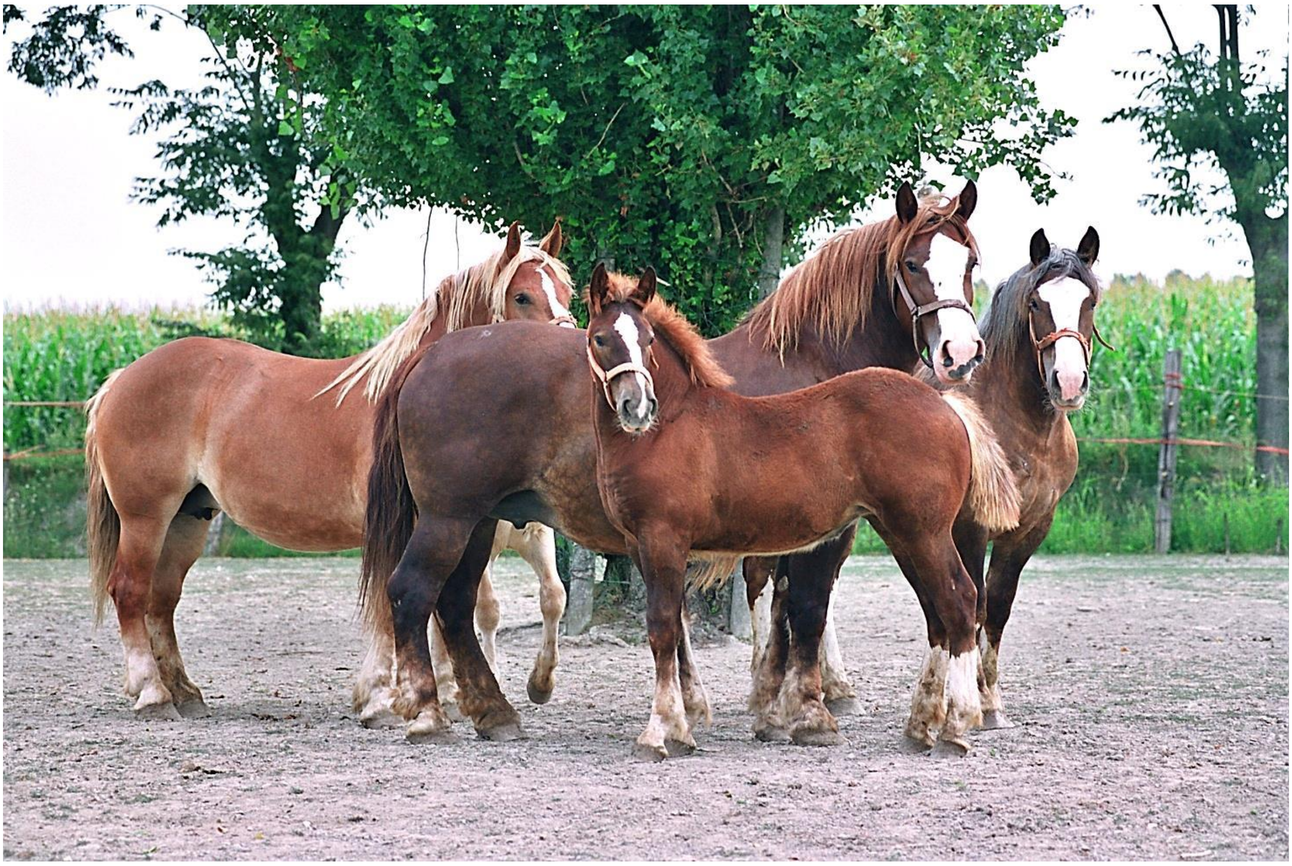

Supplementary Figure S1. Mares and foal of Italian Heavy Draught Horse (IHDH; courtesy of Cinzia Stoppa)

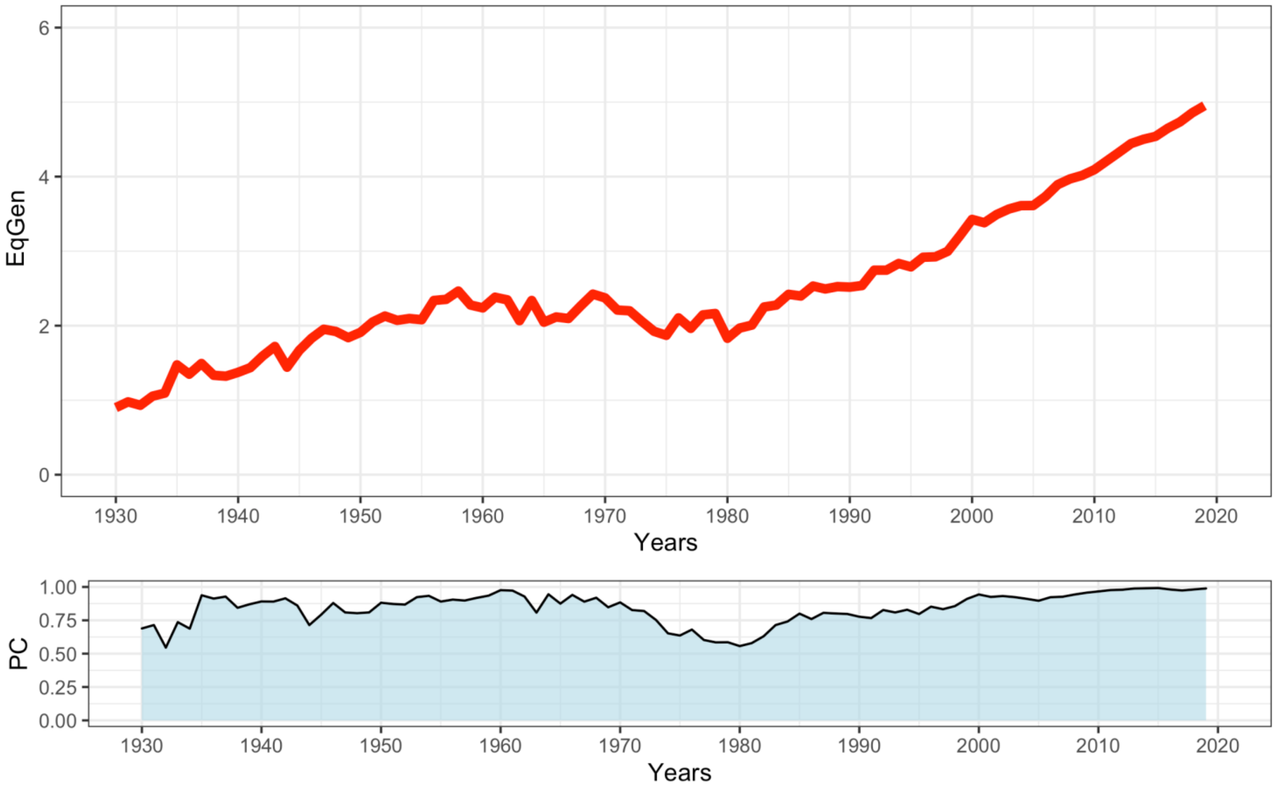

Supplementary Figure S2. Number of Equivalent Generations (EqGen; above) and pedigree completeness (PC; below) over years in Italian Heavy Draught Horse population.

Supplementary Table S1. Descriptive statistics of homozygosity (observed: Ho_obs; expected: Ho_exp; total: Ho_tot) in 267 genotyped individuals of Italian Heavy Draught Horse based on the number of homozygous genotypes.

Parameter	Mean	SD	Min	Max
Ho_obs	35,630.3	500.7	34,291	38,013
Ho_exp	35,707.8	64.0	35,010	35,740
Ho_tot	50,674.5	93.8	49,638	50,714

[1] Definitions of the methods for inbreeding are in the text.

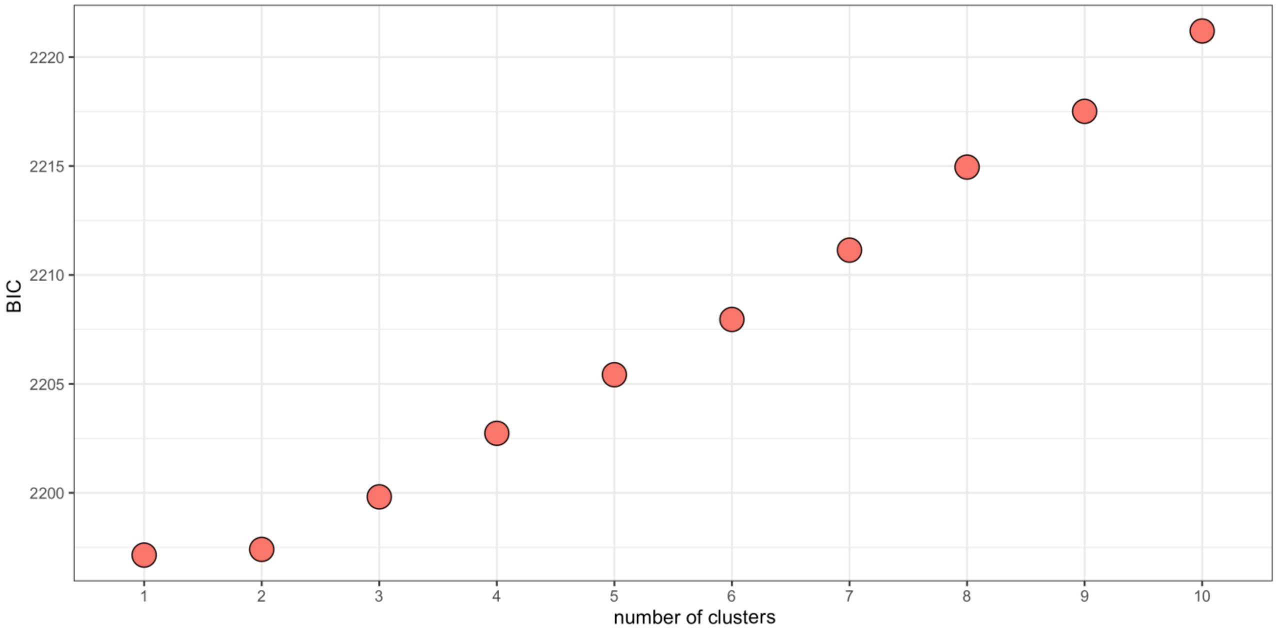

Supplementary Figure S3. Values of BIC obtained by analyzing values of K from 1 to 10, corresponding on the same amount of clusters defining the proportion of ancestry in the 267 genotyped individuals.

Supplementary Table S2. Estimation of genomic effective population size (Ne) traced back to 18 generations ago (Gen. ago). The linkage disequilibrium estimation, adjusted for sampling bias was also included (LD_r^2), as well as the relative standard deviation ($SD(LD\_r^2)$).

Gen. ago	Ne	LD_r^2	$SD(LD\_r^2)$
1	100	0.009	0.014
2	108	0.011	0.018
3	118	0.015	0.024
4	126	0.017	0.028
5	134	0.019	0.031
6	143	0.021	0.034
7	156	0.023	0.038
9	173	0.026	0.041
11	189	0.029	0.046
14	213	0.032	0.052
18	241	0.036	0.058

Supplementary Table S3. Ancestors explaining the 50% of genetic diversity in the two subpopulations (*subpop1* and *subpop2*) recognized in IHDH looking at 267 genotyped individuals

ID Individual	Cumulative contribution	Individual contribution	Name	Sex	Birth year	Origin[1]
subpop1						
25667	0.190	0.190	ISARD	M	1996	*breton*
25316	0.277	0.086	MARUSCA	F	1996	*local*
7936	0.348	0.072	OLGAN	M	1980	*breton*
22545	0.413	0.064	GONZALEZ	M	1994	*breton*
12940	0.474	0.061	VAUBAN	M	1987	*breton*
8966	0.514	0.040	QUETEUR	M	1982	*breton*
subpop2						
22545	0.081	0.081	GONZALEZ	M	1994	*breton*
8966	0.131	0.050	QUETEUR	M	1982	*breton*
12940	0.181	0.050	VAUBAN	M	1987	*breton*
25667	0.230	0.049	ISARD	M	1996	*breton*
7936	0.278	0.048	OLGAN	M	1980	*breton*
2733	0.312	0.034	LUCIFER	M	1955	*breton*
30326	0.345	0.033	KAMIKAZE 4	M	1998	*breton*
31039	0.370	0.024	LARMEL D.K.	M	1999	*breton*
11017	0.393	0.024	TILMA	M	1985	*breton*
4327	0.417	0.023	URENIO	M	1964	*local*
6746	0.437	0.020	LANDERNEAU	M	1977	*breton*
22513	0.456	0.020	IMOLA G.	F	1994	*local*
45607	0.476	0.019	ZORRO	M	2006	*local*
6521	0.494	0.019	KLEBER	M	1976	*breton*
49482	0.511	0.017	CARONI' P.A.	M	2009	*local*

[1] *local*: native IHDH animals, that is from Italy; *breton*: French Norfolk-Breton animal

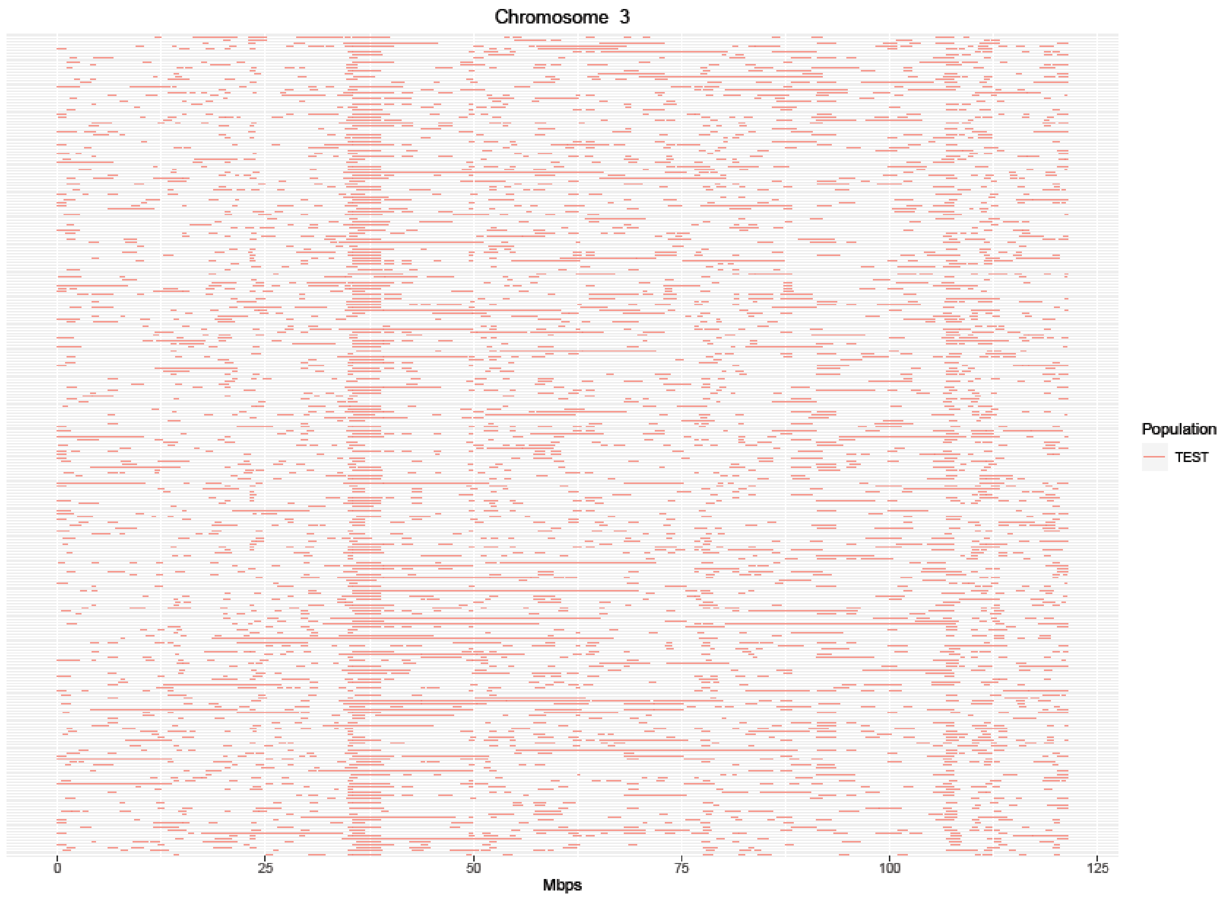

Supplementary Figure S4. Map of ROH islands identified in horse chromosome 3.

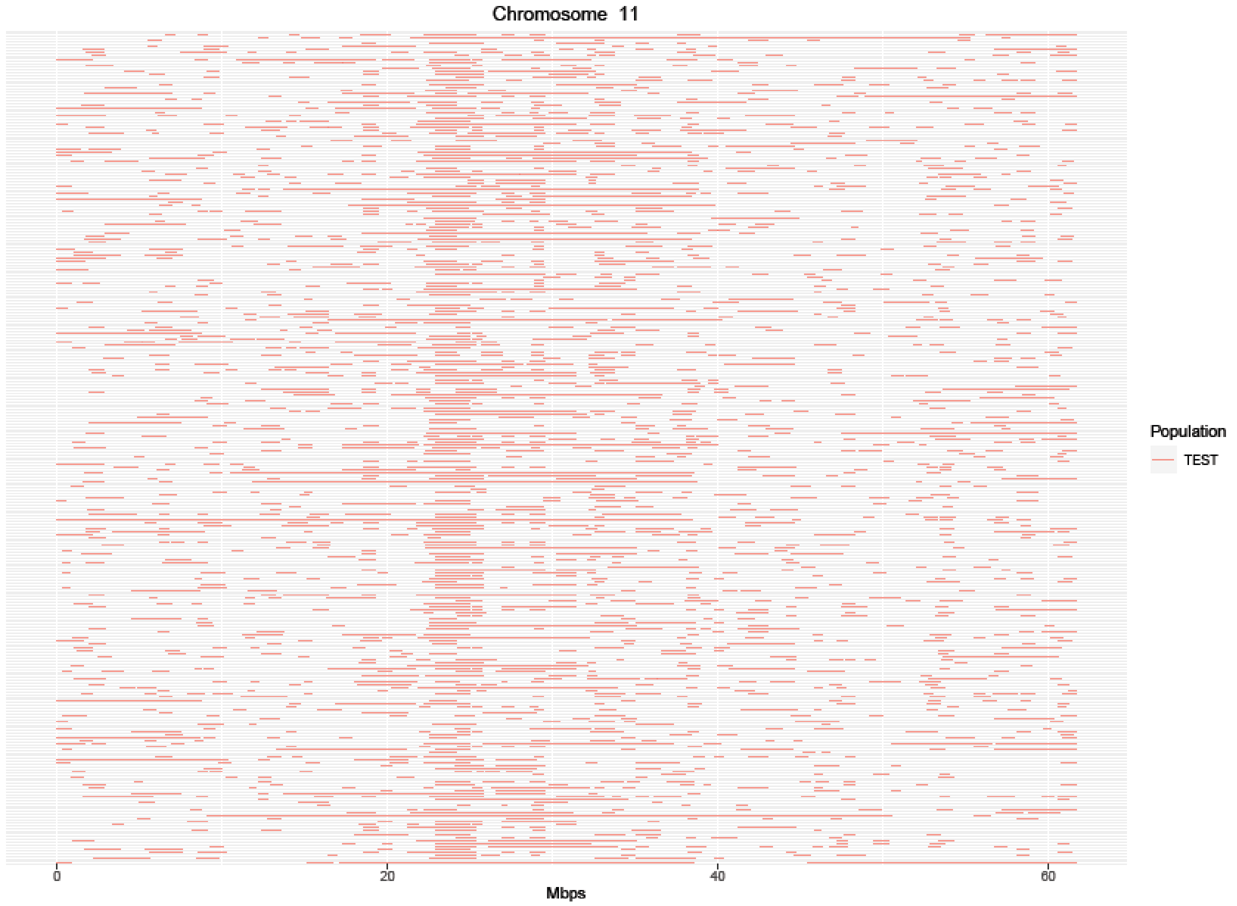

Supplementary Figure S5. Map of ROH islands identified in horse chromosome 11.

Supplementary Table S4. Genes coordinates, names and types included in the ROH islands shared in over 60% of the IHDH horses with genomic information

Gene start (bp)	Gene end (bp)	Chromosome/scaffold name	Gene name	Gene type
25399577	25423540	10	NLRP4	protein_coding
25428474	25449869	10	NLRP13	protein_coding
25471710	25502721	10	NLRP5	protein_coding
25519774	25549093	10	ZNF787	protein_coding
25562951	25578480	10	ZNF444	protein_coding
25594177	25600088	10	GALP	protein_coding
25649032	25675911	10	EDDM13	protein_coding
25720149	25739369	10	ZNF667	protein_coding
25753879	25766117	10	ZNF583	protein_coding
25780140	25795189	10	ZNF582	protein_coding
25849716	25862704	10	ZNF471	protein_coding
25869395	25894335	10	ZFP28	protein_coding
25904913	25919454	10	ZNF470	protein_coding
25942428	25943858	10	ZNF71	protein_coding
22909966	22960607	11	CDK12	protein_coding
22967229	22991769	11	MED1	protein_coding
22993134	23094694	11	FBXL20	protein_coding
23130157	23165075	11	CACNB1	protein_coding
23141809	23146463	11	RPL19	protein_coding
23171324	23176533	11	ARL5C	protein_coding
23180941	23242779	11	PLXDC1	protein_coding
23270527	23289068	11	FBXO47	protein_coding
23296915	23296992	11	LINC00672	protein_coding
23301513	23339708	11	LASP1	protein_coding
23351528	23358371	11	RPL23	protein_coding
23352223	23352356	11	SNORA21	snoRNA
23359381	23362574	11	C17orf98	protein_coding
23376956	23397909	11	CWC25	protein_coding
23399457	23423517	11	PIP4K2B	protein_coding
23428132	23593046	11	MLLT6	protein_coding
23441117	23451027	11	PCGF2	protein_coding
23452573	23455269	11	CISD3	protein_coding
23499837	23500976	11	EPOP	protein_coding
23552512	23618630	11	SRCIN1	protein_coding
23631663	23699451	11	ARHGAP23	protein_coding
23718048	23746147	11	SOCS7	protein_coding
23751506	23766135	11	GPR179	protein_coding
23769289	23813041	11	MRPL45	protein_coding
23812720	23986851	11	NPEPPS	protein_coding
23926687	23951503	11	KPNB1	protein_coding
23959585	23976549	11	TBKBP1	protein_coding
23998627	24009622	11	TBX21	protein_coding
24059477	24073469	11	OSBPL7	protein_coding
24074571	24080964	11	MRPL10	protein_coding
24080985	24085707	11	LRRC46	protein_coding
24086512	24089667	11	SCRN2	protein_coding
24095634	24097456	11	SP6	protein_coding
24109848	24109992	11	eca-mir-9103	miRNA

24136454	24161471	11		SP2	protein_coding
24170087	24174863	11		PNPO	protein_coding
24276709	24280640	11		CBX1	protein_coding
24302456	24308143	11		SNX11	protein_coding
24319010	24574312	11		SKAP1	protein_coding
24655096	24657457	11		HOXB1	protein_coding
24666844	24668929	11		HOXB2	protein_coding
24674374	24676499	11		HOXB3	protein_coding
24700383	24702012	11		HOXB4	protein_coding
24703522	24703631	11		eca-mir-10a	miRNA
24715900	24717387	11		HOXB5	protein_coding
24720005	24728090	11		HOXB6	protein_coding
24730628	24733955	11		HOXB7	protein_coding
24735330	24737767	11		HOXB8	protein_coding
24745908	24749775	11		HOXB9	protein_coding
24755448	24755517	11		eca-mir-196a	miRNA
24814487	24816968	11		HOXB13	protein_coding
24838169	24886617	11		TTLL6	protein_coding
24897772	24918067	11		CALCOCO2	protein_coding
67359774	67447936	15		LBH	protein_coding
67509458	67523395	15		YPEL5	protein_coding
67710548	68366314	15		ALK	protein_coding
35177303	35208964	3		CA5A	protein_coding
35218960	35336287	3		BANP	protein_coding
35654813	35666467	3		ZNF469	protein_coding
35728941	35748261	3		ZFPM1	protein_coding
35818916	35871819	3		ZC3H18	protein_coding
35877900	35879418	3		IL17C	protein_coding
35882283	35891561	3		CYBA	protein_coding
35893235	35901155	3		MVD	protein_coding
35915990	35921801	3		SNAI3	protein_coding
35931207	35940708	3		RNF166	protein_coding
35940159	36052594	3		CTU2	protein_coding
35949711	36015387	3		PIEZO1	protein_coding
36033038	36039482	3		CDT1	protein_coding
36039403	36041761	3		APRT	protein_coding
36043555	36071575	3		GALNS	protein_coding
36071873	36075052	3		TRAPPC2L	protein_coding
36079360	36081986	3		PABPN1L	protein_coding
36089907	36177185	3		CBFA2T3	protein_coding
36280898	36349736	3		ACSF3	protein_coding
36370076	36389221	3		CDH15	protein_coding
36424787	36557180	3		ANKRD11	protein_coding
36640217	36640361	3		eca-mir-9074	miRNA
36652428	36765799	3		SPG7	protein_coding
36690533	36693080	3		RPL13	protein_coding
36691118	36691198	3		SNORD68	snoRNA
36704238	36720241	3		CPNE7	protein_coding
36744927	36764947	3		DPEP1	protein_coding
36769405	36777049	3		CHMP1A	protein_coding
36777286	36786652	3		SPATA33	protein_coding
36788604	36797021	3		CDK10	protein_coding

36796897	36800902	3	SPATA2L	protein_coding
36806471	36817974	3	VPS9D1	protein_coding
36818474	36836378	3	ZNF276	protein_coding
36836394	36893474	3	FANCA	protein_coding
36909854	36940876	3	SPIRE2	protein_coding
36944622	37015067	3	TCF25	protein_coding

Supplementary Table S5. Genes coordinates, names and types included in the ROH islands shared in over 60% of the two subpopulations of IHDH horses with genomic information recognized after clustering analysis

Gene name	Gene type	Gene start (bp)	Gene end (bp)	Chromosome/scaffold name	Group
CA5A	protein_coding	35177303	35208964	3	1,2
BANP	protein_coding	35218960	35336287	3	1,2
ZNF469	protein_coding	35654813	35666467	3	1,2
ZFPM1	protein_coding	35728941	35748261	3	1,2
ZC3H18	protein_coding	35818916	35871819	3	1,2
IL17C	protein_coding	35877900	35879418	3	1,2
CYBA	protein_coding	35882283	35891561	3	1,2
MVD	protein_coding	35893235	35901155	3	1,2
SNAI3	protein_coding	35915990	35921801	3	1,2
RNF166	protein_coding	35931207	35940708	3	1,2
CTU2	protein_coding	35940159	36052594	3	1,2
PIEZO1	protein_coding	35949711	36015387	3	1,2
CDT1	protein_coding	36033038	36039482	3	1,2
APRT	protein_coding	36039403	36041761	3	1,2
GALNS	protein_coding	36043555	36071575	3	1,2
TRAPPC2L	protein_coding	36071873	36075052	3	1,2
PABPN1L	protein_coding	36079360	36081986	3	1,2
CBFA2T3	protein_coding	36089907	36177185	3	1,2
ACSF3	protein_coding	36280898	36349736	3	1,2
CDH15	protein_coding	36370076	36389221	3	1,2
ANKRD11	protein_coding	36424787	36557180	3	1,2
eca-mir-9074	miRNA	36640217	36640361	3	1,2
SPG7	protein_coding	36652428	36765799	3	1,2
RPL13	protein_coding	36690533	36693080	3	1,2
SNORD68	snoRNA	36691118	36691198	3	1,2
CPNE7	protein_coding	36704238	36720241	3	1,2
DPEP1	protein_coding	36744927	36764947	3	1,2
CHMP1A	protein_coding	36769405	36777049	3	1,2
SPATA33	protein_coding	36777286	36786652	3	1,2
CDK10	protein_coding	36788604	36797021	3	1,2
SPATA2L	protein_coding	36796897	36800902	3	1,2
VPS9D1	protein_coding	36806471	36817974	3	1,2
ZNF276	protein_coding	36818474	36836378	3	1,2
FANCA	protein_coding	36836394	36893474	3	1,2
SPIRE2	protein_coding	36909854	36940876	3	1,2
TCF25	protein_coding	36944622	37015067	3	1,2
DEF8	protein_coding	37004218	37013778	3	1,2
DBNDD1	protein_coding	37052595	37061108	3	1,2
GAS8	protein_coding	37065811	37082990	3	1,2
CENPE	protein_coding	37409182	37494360	3	1,2

BDH2	protein_coding	37504187	37531456	3	1,2
SLC9B2	protein_coding	37546027	37588480	3	1,2
SLC9B1	protein_coding	37595240	37698997	3	1,2
CISD2	protein_coding	37697116	37710309	3	1,2
MANBA	protein_coding	37805928	37919128	3	1,2
NFKB1	protein_coding	37926954	38031851	3	1,2
SLC39A8	protein_coding	38150557	38244356	3	1,2
BANK1	protein_coding	38353378	38664247	3	1,2
TRAF3IP1	protein_coding	24225781	24278832	6	1
ASB1	protein_coding	24303035	24320103	6	1
eca-mir-8979	miRNA	24322196	24322340	6	1
KIF1A	protein_coding	26098411	26172631	6	1
AGXT	protein_coding	26227614	26238320	6	1
MAB21L4	protein_coding	26242593	26252725	6	1
CROCC2	pseudogene	26263006	26338073	6	1
SNED1	protein_coding	26343991	26419036	6	1
MTERF4	protein_coding	26425468	26430190	6	1
PASK	protein_coding	26440026	26470519	6	1
PPP1R7	protein_coding	26476087	26506729	6	1
HDLBP	protein_coding	26548738	26585377	6	1
SEPTIN2	protein_coding	26632179	26654710	6	1
FARP2	protein_coding	26675269	26770872	6	1
WNT5B	protein_coding	30095457	30107573	6	2
FBXL14	protein_coding	30137702	30144462	6	2
ERC1	protein_coding	30219683	30698429	6	2
RAD52	protein_coding	30790724	30815021	6	2
WNK1	protein_coding	30816076	30970274	6	2
NINJ2	protein_coding	31044492	31115888	6	2
NLRP4	protein_coding	25399577	25423540	10	1,2
NLRP13	protein_coding	25428474	25449869	10	1,2
NLRP5	protein_coding	25471710	25502721	10	1,2
ZNF787	protein_coding	25519774	25549093	10	1,2
ZNF444	protein_coding	25562951	25578480	10	1,2
GALP	protein_coding	25594177	25600088	10	1,2
EDDM13	protein_coding	25649032	25675911	10	1,2
ZNF667	protein_coding	25720149	25739369	10	1,2
ZNF583	protein_coding	25753879	25766117	10	1,2
ZNF582	protein_coding	25780140	25795189	10	1,2
ZNF471	protein_coding	25849716	25862704	10	1,2
ZFP28	protein_coding	25869395	25894335	10	1,2
ZNF470	protein_coding	25904913	25919454	10	1,2
ZNF71	protein_coding	25942428	25943858	10	1,2
ZNF304	protein_coding	26641472	26689882	10	1,2
eca-mir-9089-2	miRNA	26666857	26666971	10	1,2
ZNF772	protein_coding	26838511	26844386	10	1,2
ZNF773	protein_coding	26849663	26860991	10	1,2
EQUCABV1R906	protein_coding	26885299	26886246	10	1,2
ZNF550	protein_coding	26901665	26911724	10	1,2
EQUCABV1R907	protein_coding	26933864	26934796	10	1,2
ZNF134	protein_coding	27106842	27109430	10	1,2
CDK12	protein_coding	22909966	22960607	11	1,2
MED1	protein_coding	22967229	22991769	11	1,2

FBXL20	protein_coding	22993134	23094694	11	1,2
CACNB1	protein_coding	23130157	23165075	11	1,2
RPL19	protein_coding	23141809	23146463	11	1,2
ARL5C	protein_coding	23171324	23176533	11	1,2
PLXDC1	protein_coding	23180941	23242779	11	1,2
FBXO47	protein_coding	23270527	23289068	11	1,2
LINC00672	protein_coding	23296915	23296992	11	1,2
LASP1	protein_coding	23301513	23339708	11	1,2
RPL23	protein_coding	23351528	23358371	11	1,2
SNORA21	snoRNA	23352223	23352356	11	1,2
C17orf98	protein_coding	23359381	23362574	11	1,2
CWC25	protein_coding	23376956	23397909	11	1,2
PIP4K2B	protein_coding	23399457	23423517	11	1,2
MLLT6	protein_coding	23428132	23593046	11	1,2
PCGF2	protein_coding	23441117	23451027	11	1,2
CISD3	protein_coding	23452573	23455269	11	1,2
EPOP	protein_coding	23499837	23500976	11	1,2
SRCIN1	protein_coding	23552512	23618630	11	1,2
ARHGAP23	protein_coding	23631663	23699451	11	1,2
SOCS7	protein_coding	23718048	23746147	11	1,2
GPR179	protein_coding	23751506	23766135	11	1,2
MRPL45	protein_coding	23769289	23813041	11	1,2
NPEPPS	protein_coding	23812720	23986851	11	1,2
KPNB1	protein_coding	23926687	23951503	11	1,2
TBKBP1	protein_coding	23959585	23976549	11	1,2
TBX21	protein_coding	23998627	24009622	11	1,2
OSBPL7	protein_coding	24059477	24073469	11	1,2
MRPL10	protein_coding	24074571	24080964	11	1,2
LRRC46	protein_coding	24080985	24085707	11	1,2
SCRN2	protein_coding	24086512	24089667	11	1,2
SP6	protein_coding	24095634	24097456	11	1,2
eca-mir-9103	miRNA	24109848	24109992	11	1,2
SP2	protein_coding	24136454	24161471	11	1,2
PNPO	protein_coding	24170087	24174863	11	1,2
PRR15L	protein_coding	24180105	24180407	11	1,2
CDK5RAP3	protein_coding	24194368	24203403	11	1,2
COPZ2	protein_coding	24238071	24247772	11	1,2
NFE2L1	protein_coding	24258594	24267604	11	1,2
CBX1	protein_coding	24276709	24280640	11	1,2
SNX11	protein_coding	24302456	24308143	11	1,2
SKAP1	protein_coding	24319010	24574312	11	1,2
HOXB1	protein_coding	24655096	24657457	11	1,2
HOXB2	protein_coding	24666844	24668929	11	1,2
HOXB3	protein_coding	24674374	24676499	11	1,2
HOXB4	protein_coding	24700383	24702012	11	1,2
eca-mir-10a	miRNA	24703522	24703631	11	1,2
HOXB5	protein_coding	24715900	24717387	11	1,2
HOXB6	protein_coding	24720005	24728090	11	1,2
HOXB7	protein_coding	24730628	24733955	11	1,2
HOXB8	protein_coding	24735330	24737767	11	1,2
HOXB9	protein_coding	24745908	24749775	11	1,2
eca-mir-196a	miRNA	24755448	24755517	11	1,2

HOXB13	protein_coding	24814487	24816968	11	1,2
TTLL6	protein_coding	24838169	24886617	11	1,2
CALCOCO2	protein_coding	24897772	24918067	11	1,2
ATP5MC1	protein_coding	24962511	24965533	11	1,2
UBE2Z	protein_coding	24972011	24985661	11	1,2
B4GALNT2	protein_coding	25134649	25189829	11	1
GNGT2	protein_coding	25217004	25222222	11	1
ABI3	protein_coding	25223503	25233953	11	1
PHOSPHO1	protein_coding	25233870	25240161	11	1
ZNF652	protein_coding	25279969	25293342	11	1
KIF2B	protein_coding	29092365	29096880	11	1
LBH	protein_coding	67359774	67447936	15	1,2
YPEL5	protein_coding	67509458	67523395	15	1,2
ALK	protein_coding	67710548	68366314	15	1,2
BICD2	protein_coding	54889930	54939060	23	1
IPPK	protein_coding	54972783	55020173	23	1
CENPP	protein_coding	55022512	55238104	23	1
ECM2	protein_coding	55074261	55100407	23	1
ASPN	protein_coding	55120039	55136355	23	1
OMD	protein_coding	55154210	55156012	23	1
OGN	protein_coding	55166519	55179646	23	1
NOL8	protein_coding	55239639	55262025	23	1
IARS1	protein_coding	55265144	55341383	23	1
SNORA84	snoRNA	55267754	55267886	23	1
ZNF484	protein_coding	55385363	55405148	23	1
RTN1	protein_coding	7000952	7216947	24	1
